# Influences of Anatomorphological Features of the Portal Venous System on Postsplenectomy Hemodynamic Characteristics in Patients With Portal Hypertension: A Computational Model-Based Study

**DOI:** 10.3389/fphys.2021.661030

**Published:** 2021-04-12

**Authors:** Tianqi Wang, Zunqiang Zhou, Fuyou Liang

**Affiliations:** ^1^State Key Laboratory of Ocean Engineering, School of Naval Architecture, Ocean and Civil Engineering, Shanghai Jiao Tong University, Shanghai, China; ^2^Department of Surgery, Shanghai Jiao Tong University Affiliated Sixth People’s Hospital, Shanghai, China; ^3^Key Laboratory of Hydrodynamics (Ministry of Education), School of Naval Architecture, Ocean and Civil Engineering, Shanghai Jiao Tong University, Shanghai, China; ^4^World-Class Research Center “Digital Biodesign and Personalized Healthcare,” Sechenov First Moscow State Medical University, Moscow, Russia

**Keywords:** portal hypertension, splenectomy, postsplenectomy thrombosis, risk factor, computational model, hemodynamics

## Abstract

Splenectomy, as an effective surgery for relieving complications caused by portal hypertension, is often accompanied by a significantly increased incidence of postoperative thrombosis in the portal venous system (PVS). While the underlying mechanisms remain insufficiently understood, the marked changes in hemodynamic conditions in the PVS following splenectomy have been suggested to be a potential contributing factor. The aim of this study was to investigate the influences of the anatomorphological features of the PVS on hemodynamic characteristics before and after splenectomy, with emphasis on identifying the specific anatomorphological features that make postoperative hemodynamic conditions more clot-promoting. For this purpose, idealized computational hemodynamics models of the PVS were constructed based on general anatomical structures and population-averaged geometrical parameters of the PVS. In the models, we incorporated various anatomorphological variations to represent inter-patient variability. The analyses of hemodynamic data were focused on the spatial distribution of wall shear stress (WSS) and the area ratio of wall regions exposed to low WSS (ALS). Obtained results showed that preoperative hemodynamic conditions were comparable among different models in terms of space-averaged WSS and ALS (all were small) irrespective of the considerable differences in spatial distribution of WSS, whereas, the inter-model differences in ALS were significantly augmented after splenectomy, with the value of ALS reaching up to over 30% in some models, while being smaller than 15% in some other models. Postoperative ALS was mainly determined by the anatomical structure of the PVS, followed by some morphogeometrical parameters, such as the diameter and curvature of the splenic vein, and the distance between the inferior mesenteric vein and splenoportal junction. Relatively, the angles between tributary veins and trunk veins only had mild influences on ALS. In addition, a marked increase in blood viscosity was predicted after splenectomy, especially in regions with low WSS, which may play an additive role to low WSS in initiating thrombosis. These findings suggest that the anatomical structure and some morphogeometrical features of the PVS are important determinants of hemodynamic conditions following splenectomy, which may provide useful clues to assessing the risk of postsplenectomy thrombosis based on medical imaging data.

## Introduction

Splenectomy is a surgery whose indications generally include spleen rupture, hypersplenism, and symptomatic splenomegaly (Cooper and Williamson, 1984; Weledji, 2014). In particular, splenectomy is often implemented in combination with porta-azygous devascularization to treat patients with portal hypertension and hypersplenism, which is expected to reduce the amount of venous blood flowing into the liver thereby helping lower portal pressure and improve liver function (Ikegami et al., 2008; Kawanaka et al., 2014). However, clinical studies have found that patients with histories of splenectomy are susceptible to the development of thrombosis in the portal venous system (i.e., portal venous system thrombosis (PVST)), with the morbidity ranging from 7 to 55% (Ikeda et al., 2005; Ushitora et al., 2011), an incidence much higher than that (approximately 5% to10%) in all cases of portal hypertension (Parikh et al., 2010). Given the severe complications (e.g., acute hypertension in splanchnic circulation and intestinal infarct) and high mortality secondary to PVST, assessing the risk of postsplenectomy thrombosis is crucial to patient management in the clinical workflow of splenectomy (Winslow et al., 2002; Ruiz-Tovar and Priego, 2016).

The pathogenesis of venous thrombosis involves multiple factors, among which blood constituents and hemodynamic factors are most frequently concerned (Rosendaal, 1993; Rasche, 2001). With regard to postsplenectomy thrombosis, the roles of blood constituents have been investigated extensively (Sobhonslidsuk and Reddy, 2002; Kinjo et al., 2010; Ruiz-Tovar and Priego, 2016). Major findings in this direction include: (1) thrombophilic disorders (e.g., deficiencies of protein C, protein S, and antithrombin III, myeloproliferative disorders, and antiphospholipid syndrome) were associated with PVST, and (2) low white cell count or high platelet count might increase the risk of postsplenectomy thrombosis. However, controversies remain about whether these factors are able to sufficiently account for the clinically observed inter-patient differences in the risk of postsplenectomy thrombosis (Winslow et al., 2002; Tsamalaidze et al., 2018). In this context, some studies turned to investigating other factors that are related to hemodynamic conditions in the portal venous system (PVS). For instance, it was found that patients with a larger diameter of the splenic vein (SV) or the portal vein (PV) had a higher risk of developing postsplenectomy thrombosis (Danno et al., 2009; Kinjo et al., 2010; de’Angelis et al., 2017; Huang et al., 2018). The study by Huang et al. (2018) further demonstrated that a higher preoperative flow rate in the PV was an independent risk factor of postsplenectomy thrombosis. The correlations of these factors with the risk of postsplenectomy thrombosis have been considered to be mediated by their influences on hemodynamic characteristics (i.e., flow disturbance or flow stagnation) that increase coagulation ability in the PVS after splenectomy (Winslow et al., 2002). Unfortunately, no existing clinical studies provided details on how blood flow patterns in the PVS are altered by splenectomy to facilitate the development of thrombosis. Nonetheless, relevant evidences have been reported by studies on thrombotic problems in other vessels or implantable artificial devices (Malek et al., 1999; Corbett et al., 2010; Gorring et al., 2015; Poredos and Jezovnik, 2018), among which, low wall shear stress (WSS), which can induce endothelial dysfunction and promote focal platelet aggregation and fibrin deposition, has been demonstrated to be a major driving factor for thrombosis.

From the hemodynamic point of view, splenectomy, due to the removal of blood flow from the spleen, would induce a marked decrease in blood flow and lowering of WSS in the PVS. In addition, flow patterns in the PVS could be highly patient-specific given the inter-patient variability in vascular anatomorphology, a major determinant of local flow patterns (Li et al., 2019; Zhou et al., 2020). Previous studies have shown that the anatomical structure (characterized mainly by the connecting positions of tributaries) of the PVS exhibits evident variations in the population, and their morphogeometrical features, such as the angle, diameter, or curvature, also differ considerably among patients (Zhang et al., 2009; Khamanarong et al., 2016). Therefore, it would be interesting to investigate how the anatomorphological features of the PVS affect hemodynamic conditions in the PVS following splenectomy, which may not only provide biomechanical evidence for explaining relevant clinical findings but also gain useful insights for assessing the risk of postsplenectomy thrombosis based on medical images of the PVS.

Given the fact that *in vivo* measurement of the details of blood flow patterns and high-precision quantification of hemodynamic parameters (e.g., WSS) in the PVS remain challenging in the clinical settings, computational hemodynamics modeling may serve as an alternative approach. In the literature, computational models have been widely used to address hemodynamic problems related to the diagnosis of portal hypertension (Wang et al., 2017, 2018, 2020), the pathogenesis or surgical treatment of liver diseases (Ho et al., 2013; Peeters et al., 2015; Audebert et al., 2018; Golse et al., 2020), or the presence of thrombus in the PVS (Petkova et al., 2003; Wang et al., 2014; Zhou et al., 2015; Aktar and Islam, 2017), but no models have been applied to address the hemodynamic impacts of splenectomy. In the present study, we built computational models to simulate hemodynamics in the PVS before and after splenectomy. The modeling work started from building a baseline idealized model of the PVS based on population-averaged anatomical and geometrical data reported in previous clinical studies, and further incorporated various variations in anatomy or morphogeometrical features to yield a series of models that represent inter-patient variability, thereby establishing a basis for identifying, through hemodynamic analyses, the specific anatomorphological features of the PVS that render postsplenectomy hemodynamic conditions clot-promoting.

## Materials and Methods

### Construction of Baseline Geometrical Models of the PVS Before and After Splenectomy

The anatomical structure of the PVS has considerable variations among individuals. In general, the PVS consists of the PV, SV, superior mesenteric vein (SMV), and other tributaries, among which the relatively large tributaries are the left gastric vein (LGV) and inferior mesenteric vein (IMV). Anatomically, the SMV always connects to the splenoportal junction, but the connecting positions of the LGV and IMV vary among individuals (Khamanarong et al., 2016). For instance, blood flow from the LGV may drain into the SV (i.e., LGV-SV connection), or into the PV (i.e., LGV-PV connection); while blood flow from the IMV may drain into the SV (i.e., IMV-SV connection), or into the SMV (i.e., IMV-SMV connection) (Purcell et al., 1951; Graf et al., 1997; Zhang et al., 2007; Sakaguchi et al., 2010; Zhou et al., 2014; Khamanarong et al., 2016). The probabilities of these connections differ among populations in different regions (see the data summarized in Table 1).

**TABLE 1:** Proportions of IMV and LGV connecting positions in populations from different regions.

**Connection**	**Proportion**		**Region**	**References**
IMV-SV	56%	28/54	America	Graf et al., 1997
	68.5%	63/92	Japan	Sakaguchi et al., 2010
	55.45%	117/211	Thailand	Khamanarong et al., 2016
	45%	86/191	China	Zhang et al., 2007
	28%	28/100	America	Purcell et al., 1951
IMV-SMV	26%	14/54	America	Graf et al., 1997
	18.5%	17/92	Japan	Sakaguchi et al., 2010
	43.13%	91/211	Thailand	Khamanarong et al., 2016
	37%	71/191	China	Zhang et al., 2007
	53%	53/100	America	Purcell et al., 1951
LGV-SV	70.97%	44/62	China	Zhou et al., 2014
	46.3%	44/95	Japan	Sakaguchi et al., 2010
	20.85%	44/211	Thailand	Khamanarong et al., 2016
	27%	27/100	America	Purcell et al., 1951
LGV-PV	29.03%	18/62	China	Zhou et al., 2014
	39%	37/95	Japan	Sakaguchi et al., 2010
	77.73%	164/211	Thailand	Khamanarong et al., 2016
	67%	67/100	America	Purcell et al., 1951

*IMV, inferior mesenteric vein; SV, splenic vein; SMV, superior mesenteric vein; LGV, left gastric vein; PV, portal vein.*

**FIGURE 1:**
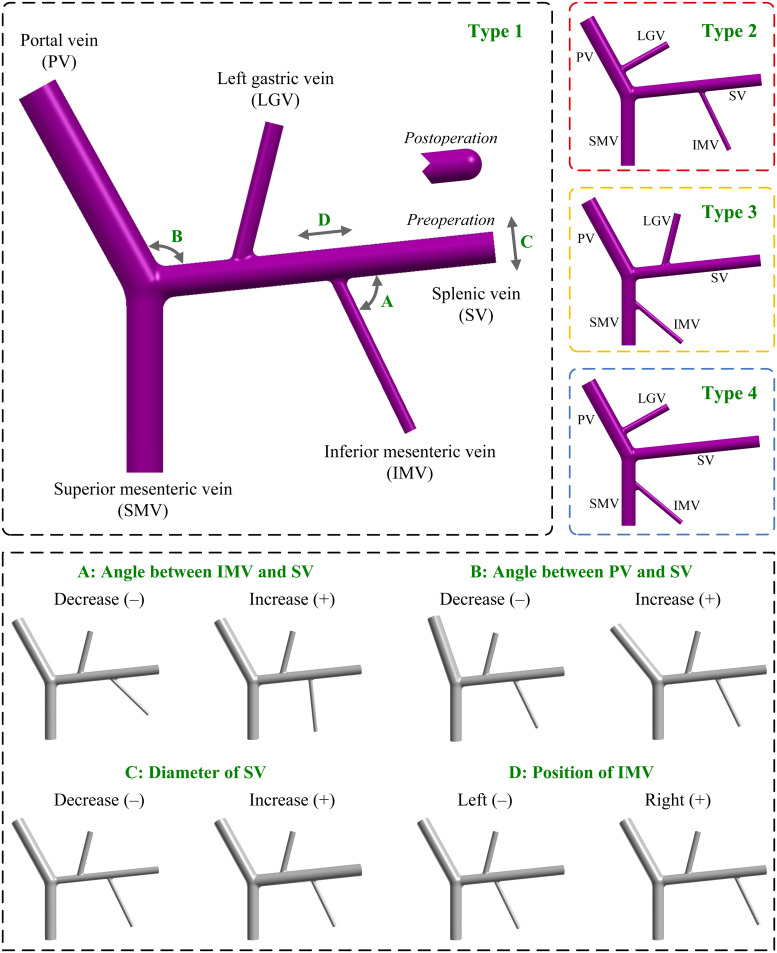
Geometrical models of the PVS with different anatomical and morphogeometrical variations. The top left panel shows the baseline anatomical structure (type 1), where the four morphogeometrical features (i.e., ‘A’, ‘B’, ‘C’, and ‘D’) are denoted. The top right panels show the three additional types (i.e., type 2, type 3, and type 4) of anatomical structure differentiated by the connecting positions of the LGV and IMV. The bottom panel illustrates the models after incorporating each of the four morphogeometrical variations. A decrease/increase in the model parameter corresponding to each of the four morphogeometrical features is denoted by (–)/(+). PV, portal vein; SV, splenic vein; SMV, superior mesenteric vein; LGV, left gastric vein; IMV, inferior mesenteric vein.

In consideration of the fact that splenectomy is a frequently adopted treatment option for patients with portal hypertension and hypersplenism in China and Japan (Qi et al., 2016), herein, we chose the anatomical structure with IMV-SV and LGV-SV connections (which have higher appearance in the populations of China and Japan), as the baseline structure (herein named as type 1), and, accordingly, an idealized computational geometrical model of the PVS was constructed and set as the baseline model (see Figure 1). In the model, the angles between the PV and vertical plane, PV and SV, and SV and SMV were set to 29°, 113°, and 96°, respectively, based on the data reported in the literature (Sztika et al., 2011). For the angle between the SV and LGV/IMV, and the distance between the splenoportal junction and LGV/IMV, relevant clinical data were absent and were herein estimated empirically according to medical images reported in the literature (Sakaguchi et al., 2010). The diameters and lengths of the PV and its tributaries in the baseline model were assigned based on the mean values of the data reported in different clinical studies (Gilfillan, 1950; Purcell et al., 1951; Matsutani et al., 1993; Ito et al., 2000; Zhang et al., 2007, 2009; Zhou et al., 2014; Wei et al., 2017) (see Table 2). The baseline model, by default, represents the intact PVS before splenectomy where the proximal end of the SV is open to blood flow from the spleen, and it was modified to represent the postsplenectomy PVS by closing the proximal end of the SV (see Figure 1) to mimic the ligation of the residual SV near the hilus of spleen after surgical resection of the spleen.

**TABLE 2:** Diameters and lengths of the PV, SV and large tributaries.

**Vessel**	**Diameter (mm)**			**Length (mm)**		
	**Literature**	**References**	**Mean**	**Literature**	**References**	**Mean**
PV	12.9 ± 2.6	Zhou et al., 2014	13.42	70	Gilfillan, 1950; Purcell et al., 1951	71.7
	13.3 ± 3.3	Ito et al., 2000		73.4		
	13.9 ± 3.3	Zhang et al., 2009				
	12.9	Wei et al., 2017				
	14.1 ± 2.5	Zhang et al., 2007				
SV	9.3 ± 2.2	Zhou et al., 2014	10.12	122	Gilfillan, 1950; Purcell et al., 1951	113.5
	10.2 ± 2.8	Ito et al., 2000		105		
	10.3 ± 3.2	Zhang et al., 2009				
	10.0	Wei et al., 2017				
	10.8 ± 2.3	Zhang et al., 2007				
SMV	12.3 ± 3.6	Ito et al., 2000	11.875	60.8	Purcell et al., 1951	60.8
	14.5 ± 3.2	Zhang et al., 2009				
	8.6	Wei et al., 2017				
	12.1 ± 2.7	Zhang et al., 2007				
LGV	6.0 ± 3.2	Zhou et al., 2014	6.0	50	Matsutani et al., 1993	50
IMV	4.1 ± 0.6	Zhang et al., 2007	4.1	59.7	Purcell et al., 1951	59.7

*It is noted that the diameter values were determined by measurements in portal hypertensive patients, whereas the length values were derived from population-averaged data reported in the literature, with the assumption that vascular lengths are similar between normal cohort and portal hypertensive patients. PV, portal vein; SV, splenic vein; SMV, superior mesenteric vein; LGV, left gastric vein; IMV, inferior mesenteric vein.*

### Incorporation of Anatomorphological Variations

Anatomorphological variations of the PVS were incorporated into the baseline model to represent inter-patient variability by means of modifying the anatomical structure or main morphogeometrical features (e.g., the angles between the SV and PV/IMV, the diameter and curvature of the SV, and the distance between the IMV and splenoportal junction). It is noted that each modification only involved one anatomical structure or one morphogeometrical feature, and that the same modification was simultaneously applied to the preoperative model and its postoperative counterpart, which would enable single factor variation analyses in hemodynamic studies to quantify the influence of each individual factor, thereby ranking the relative importance of factors to hemodynamic parameters of concern.

#### Variations of Anatomical Structure

Variations in the connecting positions of LGV and IMV represent the major anatomical variations of the PVS observed in the population. In this study, we considered all the connections listed in Table 1 when building computational models, and as such obtained three additional types of model with different anatomical structures. Herein, the type 2 model had the connections of IMV-SV and LGV-PV; the type 3 model had the connections of IMV-SMV and LGV-SV; and the type 4 model had the connections of IMV-SMV and LGV-PV. It is noted that in the three additional types of model all geometrical parameters were maintained the same as those in the baseline model, and that the distances to junctions and angles of the LGV and IMV relative to the SV, SMV or PV were estimated based on the literature data (Sakaguchi et al., 2010). The upper panels of Figure 1 illustrate the baseline model (type 1) and the three variations (i.e., type 2, type 3, and type 4).

#### Variations of Morphogeometrical Features

The main morphogeometrical features (i.e., ‘A’, ‘B’, ‘C’, and ‘D’ marked on the baseline model shown in Figure 1) of the PVS were each varied by decreasing (−) or increasing (+) the corresponding model parameter relative to the reference value (i.e., that assigned to the baseline model). Herein, ‘A’ represented the angle between the IMV and SV, which was varied by ±20° relative to the reference value (70°). ‘B’ represented the angle between the PV and SV, which was varied by ±10° relative to the reference value (113°). ‘C’ represented the diameter of the SV, which was varied by ±2.625 mm [the mean value of the standard deviations derived from the literature (Ito et al., 2000; Zhang et al., 2007, 2009; Zhou et al., 2014)] relative to the reference value (10.12 mm). ‘D’ represented the position of the IMV, which was quantitatively expressed by the distance between the IMV and splenoportal junction, and was varied by ±20 mm relative to the reference value (60 mm).

**FIGURE 2:**
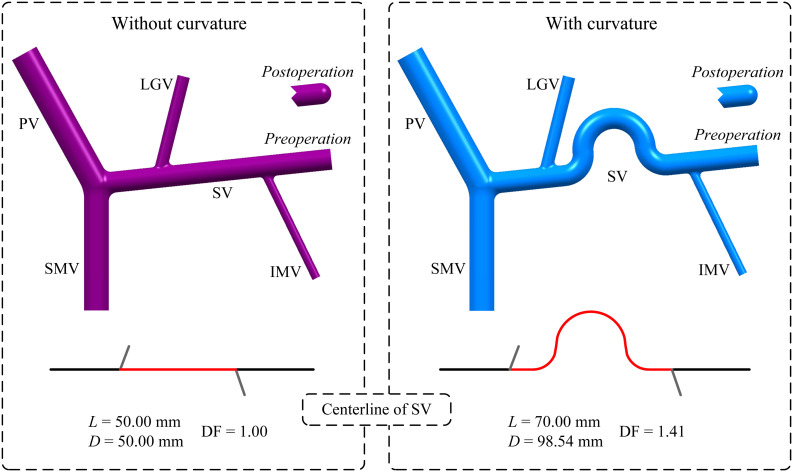
Geometrical models of the PVS and centerlines of the SV with or without curvature in the mid-SV segment between the IMV and LGV. It is noted that the model without curvature is the baseline model with ‘D’(+) variation. The red portion highlighted on each centerline represents the centerline of the mid-SV segment, for which the distance factor (DF) is calculated based on the curve length (*L*) and distance between the two terminals (*D*) (the values of DF, *L* and *D* are given) to evaluate the degree of curvature.

In addition, curvature of the SV was also incorporated by introducing a curved SV segment between the LGV and IMV in the baseline model with the ‘D’(+) variation. As shown in Figure 2, the original straight mid-SV segment between the LGV and IMV (length: 50 mm) was replaced by a curved segment whose centerline consisting of two straight lines (length: 10 mm) and three tangential arcs [i.e., two quadrants (radius: 10 mm) and one semicircle (radius: 15 mm)]. The curvature of the mid-SV segment was evaluated by the distance factor (DF) of its centerline, which was calculated as the ratio of the curve length (*L*) to the distance between the two terminals (*D*). It is noted that the diameter of the curved SV segment was maintained the same as that of the straight one.

### Mesh Generation and Setup of Hemodynamic Model

The geometrical models were each read into ICEM CFD (ANSYS Inc., United States) to generate a mesh model to be used for hemodynamic simulation. In order to improve the accuracy of hemodynamic computation in the near-wall zone, a hybrid meshing strategy was adopted where the core region of fluid domain was divided with tetrahedral elements while the near-wall region with prism elements. The minimum size of tetrahedral elements was set to 0.08 mm, and the thickness of the first near-wall prism layer was set to 0.05 mm. Numerical tests performed on the preoperative baseline model revealed that further reducing the element sizes by 20% induced less than 0.2% change in computed space-averaged WSS. Therefore, the adopted element sizes were considered to be acceptable from the numerical point of view, and applied to all models.

The inflow boundaries of the SV and tributaries in each model were set as flow velocity inlets. A parabolic velocity profile (generated based on an assigned cross-sectional mean axial velocity) was imposed at each inlet. The mean flow velocity values assigned to the inlets of SV, SMV, LGV, and IMV were derived from the population-averaged data measured in patients with portal hypertension, and were set to 8.89 cm/s (George, 2008), 5.02 cm/s (George, 2008), 7.8 cm/s (Matsutani et al., 1993), and 8 cm/s (Matsutani et al., 1993; Maruyama et al., 2013), respectively. For purpose of simplicity, all flow velocities imposed at the model inlets were assumed to be constant in consideration of the weak pulsation of blood flow in the PVS. The outflow boundary of the PV was set as a pressure outlet with the pressure being fixed at 25 mmHg, a common portal venous pressure in portal hypertensive patients (Lebrec et al., 1997). Note that the same PV outlet pressure was assigned to the preoperative and postoperative models, which, strictly speaking, is not physiologically reasonable since splenectomy will induce a decrease of portal pressure. This simplification, however, would not compromise the validity of the computation of hemodynamic parameters (e.g., WSS) of concern in the present study, which are determined by inflow rates from the SV and tributaries and anatomorphological features of the PVS rather than blood pressure at the PV outlet.

The venous walls in each model were assumed to be rigid to which the no-slip boundary condition was imposed. The assumption was reasonable because the close-to-steady state of blood flow in the PVS would not cause large displacements of venous wall. Venous blood was modeled as an incompressible non-Newtonian fluid with a density of 1,060 kg/mł, and blood flow was governed by the continuity and Navier-Stokes equations. Herein, the non-Newtonian rheology of blood was considered since blood flow in the PVS is slow, making the shear-rate dependent effect of blood viscosity more evident than in large arteries where blood flow velocities are much higher (Ho et al., 2012). In this study, the Carreau model was employed to represent the change in blood viscosity (μ) with shear rate ($\overset{\cdot }{\gamma }$),

(1) $$\mu =\mu_{\infty }+\left(\mu_{0}-\mu_{\infty }\right)\,{\left[1+{\left(\lambda \,\overset{\cdot }{\gamma }\right)}^{2}\right]}^{\left(n-1\right)/2},$$

where λ = 3.313 s, *n* = 0.3568, *μ*_0_ = 0.056 Pa⋅s, and *μ*_∞_ = 0.00345 Pa⋅s (Johnston et al., 2004).

### Numerical Simulation and Data Analysis

The governing equations of blood flow were numerically solved using a finite volume method-based commercial CFD (Computational Fluid Dynamics) package, Fluent (ANSYS Inc., United States). Herein, unsteady numerical schemes were adopted to improve numerical stability, although the boundary conditions of the models were prescribed with constant values. The time step was fixed at 0.01 s, and each model was continuously run for 3 s to eliminate the influence of uncertainty in artificially assigned initial conditions. The computed results of each model at the last time step were extracted and analyzed to derive hemodynamic parameters of interest.

In light of the fact that low WSS is closely associated with the risk of venous thrombosis (Malek et al., 1999; Poredos and Jezovnik, 2018), in this study, hemodynamic conditions in the PVS were quantitatively evaluated in terms of the spatial distribution of WSS, space-averaged WSS (SA-WSS), and the area ratio of wall regions exposed to low WSS (ALS).

(2) $$\text{SA-WSS}=\frac{1}{A_{\text{W}}}\,\sum_{i=1}^{N}{\text{WSS}}_{i}\cdot \Delta \,A_{i},$$

(3) $$\text{ALS}={\frac{1}{A_{\text{W}}}\,\sum_{i=1}^{M}\Delta \,A_{i}|}_{{\text{WSS}}_{i}<{\text{WSS}}_{\text{T}}}\times 100\%,$$

Here, *A*_W_ is the total wall area of the PVS. *ΔA_i_* is the area of the *i*th mesh surface on the PVS wall, with the corresponding WSS being denoted as WSS*_i_*. The PVS wall contains a total number of *N* mesh surfaces, among which *M* mesh surfaces were exposed to WSSes lower than the threshold value WSS_T_. Herein, WSS_T_ was set to 0.1 Pa according to the threshold value of low WSS suggested in the literature (Corbett et al., 2010; Gorring et al., 2015). The aforementioned hemodynamic parameters were computed for all models and compared in relation to the anatomorphological features of PVS.

## Results

### Comparison of Simulated Hemodynamic Parameters Among Models With Different Anatomical Variations

**FIGURE 3:**
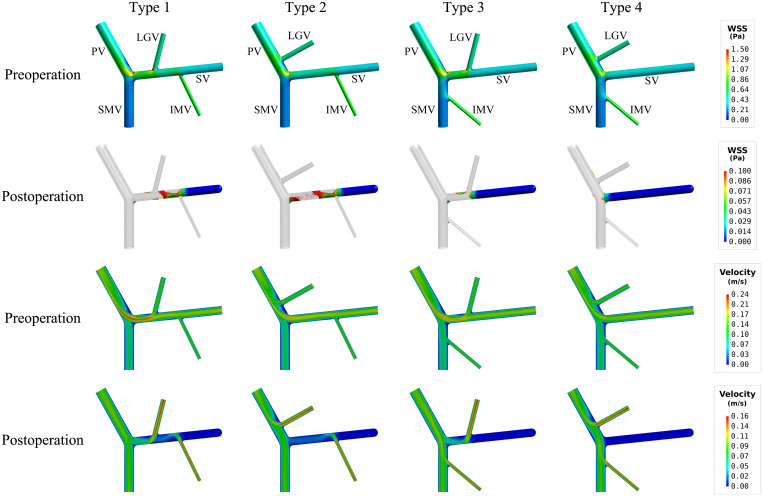
Simulated spatial distributions of WSS (the first two rows) and mid-plane contours of flow velocity (the last two rows) in the models with four types of anatomical structure. It is noted that for the postoperative models only the wall areas exposed to low WSS [i.e., lower than the threshold value (0.1 Pa)] are highlighted.

The simulated spatial distributions of WSS for the models with four types of anatomical structure before and after splenectomy are shown in Figure 3, with the corresponding values of SA-WSS and ALS being reported in Table 3. It was observed that the preoperative WSS differed considerably in spatial distribution among the models, however, the values of SA-WSS and ALS were comparable (e.g., the values of ALS computed for all the models were smaller than 1%). After splenectomy, SA-WSS decreased significantly in all the models and did not show evident inter-model differences. In contrast, ALS increased remarkably, along with evidently augmented inter-model differences. For instance, the type 4 model had the largest postoperative ALS (35.19%), followed by the type 3 model (26.48%) and type 2 model (23.06%), while the type 1 model had the smallest ALS (21.28%). The inter-model differences in postoperative ALS corresponded well with the model-simulated postoperative spatial distributions of WSS, where the type 3 and type 4 models exhibited larger wall areas exposed to WSSes lower than the threshold value (0.1 Pa), especially in the SV.

**TABLE 3:** Simulated results for models with four types of anatomical structure.

**Type**	**Condition**	**SA-WSS (Pa)**	**ALS (%)**
Type 1	Pre	0.469	0.74
	Post	0.252	21.28
Type 2	Pre	0.443	0.99
	Post	0.236	23.06
Type 3	Pre	0.450	0.73
	Post	0.237	26.48
Type 4	Pre	0.427	0.86
	Post	0.215	35.19

*For each type of anatomical structure, the simulated results before (Pre) and after (Post) splenectomy are both provided. SA-WSS, space-averaged wall shear stress; ALS, area ratio of wall regions exposed to low wall shear stress.*

The mid-plane contours of flow velocity shown in lower panels of Figure 3 indicated that the proximal segments of the SVs in the type 3 and type 4 models, due to limited blood flow draining from the tributaries, suffered from lower flow velocity after splenectomy, which can account for the enlarged area of SV wall exposed to low WSS. Before splenectomy, blood flow from the spleen is a major factor for maintaining a normal level of WSS in the SV.

### Influences of Morphogeometrical Variations on Hemodynamic Parameters

Table 4 lists the model-simulated values of SA-WSS and ALS for models with various morphogeometrical variations. ‘A’–‘D’ variations (see Figure 1 and Section “Variations of Morphogeometrical Features’ for details of their definitions) induced detectable while mild changes in SA-WSS and ALS before splenectomy, and the changes in SA-WSS remained mild after splenectomy. However, the changes in ALS were significantly enlarged after splenectomy, especially in the cases of ‘C’ and ‘D’ variations. Quantitatively, the simulated postoperative ALS values for the models with ‘A’ and ‘B’ variations fell in a narrow range from 20.64% to 21.54%, which were close to the postoperative ALS (21.28%) of the baseline model, indicating that ALS is not significantly affected by ‘A’ or ‘B’ variations. Relatively, ‘C’ and ‘D’ variations induced much larger changes in ALS. In particular, ‘C’(−) (i.e., reducing the diameter of SV by 2.625 mm) led postoperative ALS to decrease to 13.65%, while ‘C’(+) (i.e., increasing the diameter of SV by 2.625 mm) caused postoperative ALS to rise up to 29.39%.

**TABLE 4:** Simulated results for models with different morphogeometrical variations.

**Variation**	**Condition**	**SA-WSS (Pa)**	**ALS (%)**
‘A’: Angle between IMV and SV	(−) Pre	0.468	0.69
	(−) Post	0.248	21.54
	(+) Pre	0.469	0.79
	(+) Post	0.253	20.64
‘B’: Angle between PV and SV	(−) Pre	0.475	0.62
	(−) Post	0.251	21.29
	(+) Pre	0.463	0.70
	(+) Post	0.252	21.32
‘C’: Diameter of SV	(−) Pre	0.492	0.60
	(−) Post	0.290	13.65
	(+) Pre	0.471	0.84
	(+) Post	0.226	29.39
‘D’: Position of IMV	(−) Pre	0.463	0.90
	(−) Post	0.244	25.12
	(+) Pre	0.473	0.66
	(+) Post	0.258	15.79
Curving of SV	Pre	0.514	2.81
	Post	0.237	23.56

*For each morphogeometrical feature, the variation represented by a decrease/increase in the corresponding model parameter is denoted by (−)/(+).*

**FIGURE 4:**
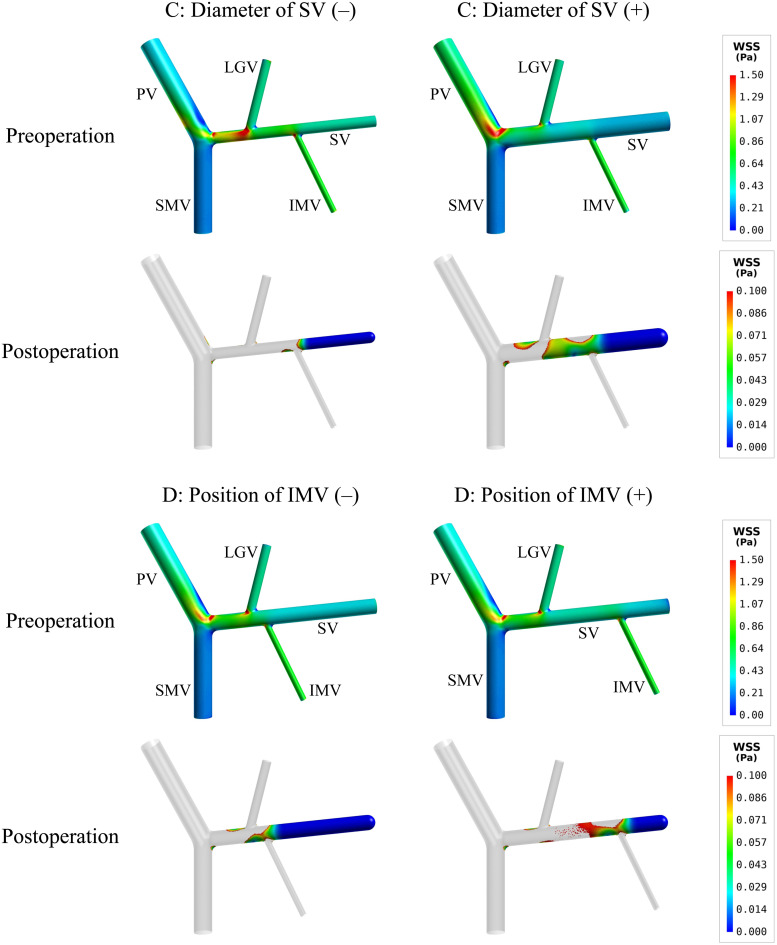
Simulated spatial distributions of WSS in the models with the variations of two representative morphogeometrical features (i.e., ‘C’ and ‘D’ variations). It is noted that for the postoperative models only the wall areas exposed to low WSS are highlighted.

**FIGURE 5:**
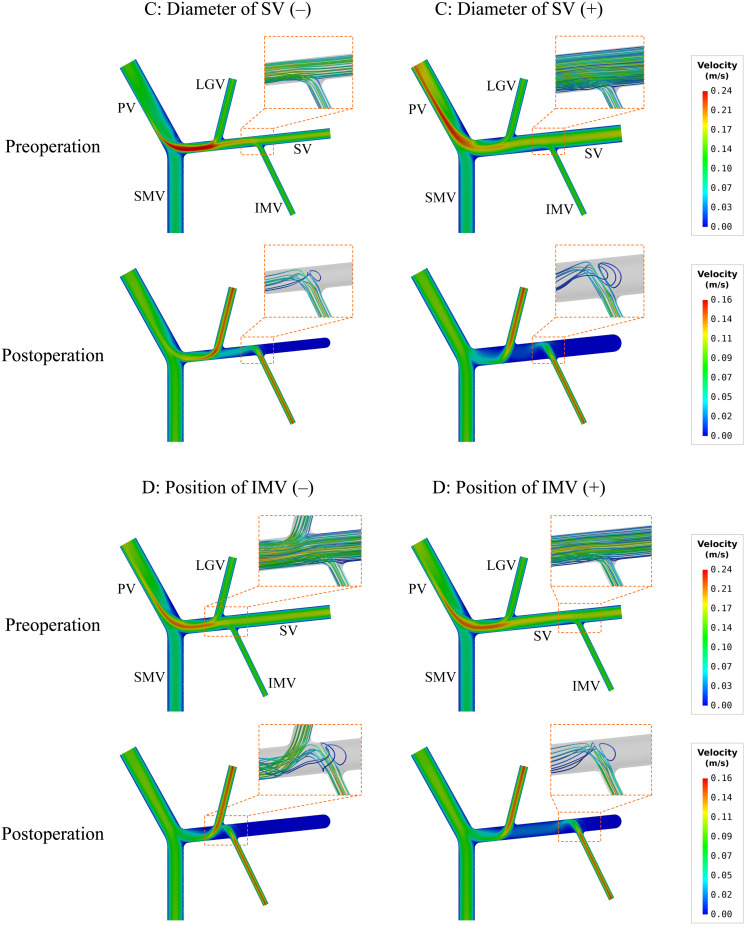
Simulated contours of flow velocity in the mid-planes of the models with the variations of two representative morphogeometrical features (i.e., ‘C’ and ‘D’ variations). The orange dashed squares highlight the characteristics of local flow fields near junctions using flow streamlines.

To further explore hemodynamic phenomena behind the data presented in Table 4, we visualize the simulated spatial distributions of WSS and flow velocity in the models with ‘C’ and ‘D’ variations in Figures 4, 5, respectively. It was observed that the distributions of WSS changed considerably in response to the incorporation of ‘C’ or ‘D’ variations both before and after splenectomy (see Figure 4). In particular, there were marked changes in wall regions exposed to low WSS (<0.1 Pa) after splenectomy. For instance, the values of WSS in the mid-SV segment located between the IMV and LGV were overall higher than the threshold value of low WSS (0.1 Pa) in the case of ‘C’(−), but decreased below 0.1 Pa in the case of ‘C’(+). In the cases of ‘D’(−) and ‘D’(+), WSS in the SV segment proximal to the IMV was most significantly affected, with ‘D’(−) inducing an enlargement of SV wall regions exposed to low WSS, while ‘D’(+) reducing the low-WSS wall regions. From the contours of flow velocity in the mid-planes of these models (see Figure 5), flow velocity in the SV before splenectomy was dominated by blood flow from the spleen, and remained high and stable in spite of variations in the diameter of SV or the connecting position of IMV. After splenectomy, the removal of blood flow from the spleen rendered blood flow in the SV highly dependent on blood perfusions from the IMV and LGV, making the flow field susceptible to disturbance at the junctions (indicated by the local streamlines) and associated distribution of low WSS sensitive to variations in the diameter of SV or the position of IMV.

**FIGURE 6:**
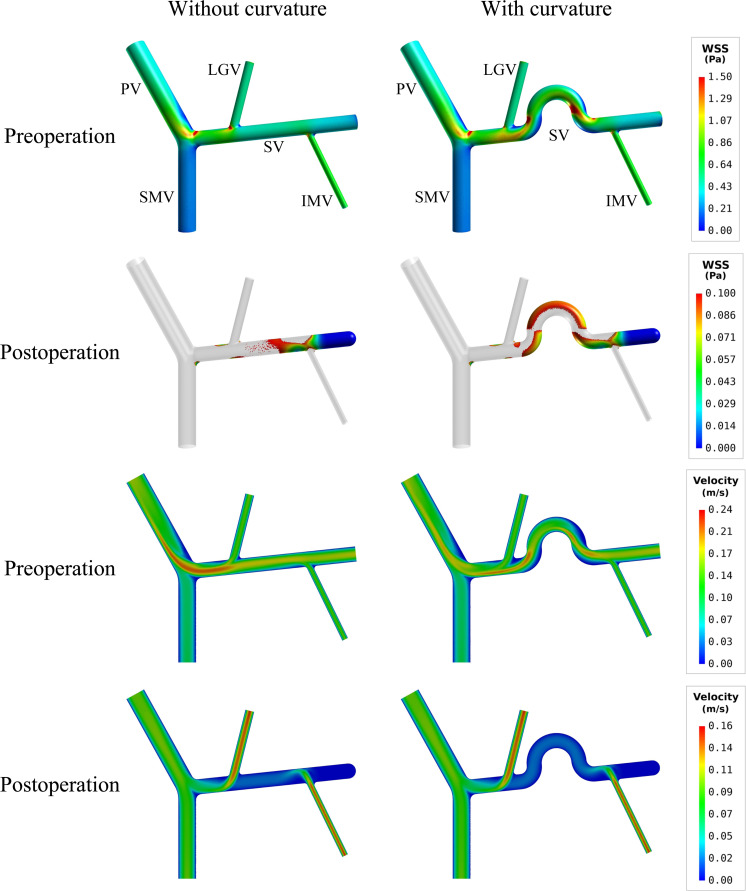
Comparison of the simulated spatial distributions of WSS and mid-plane contours of flow velocity in the models with and without curving of the mid-SV segment.

If curvature was introduced to the mid-SV segment (located between the IMV and LGV) in the model with ‘D’(+) variation (see Figure 2), simulated postoperative ALS increased from 15.79% (in the case of straight SV) to 23.56% (see Table 4). From the simulated spatial distributions of WSS and mid-plane contours of flow velocity presented in Figure 6, curving of the mid-SV segment led to marked hemodynamic changes in both the preoperative and postoperative models. A major change was the appearance of slow-flow zones near the outer walls of the curvatures, leading to locally lowering of WSS. The effects were especially strong in the postoperative model, causing a marked enlargement of the wall area subject to WSS lower than the threshold value (0.1 Pa).

**FIGURE 7:**
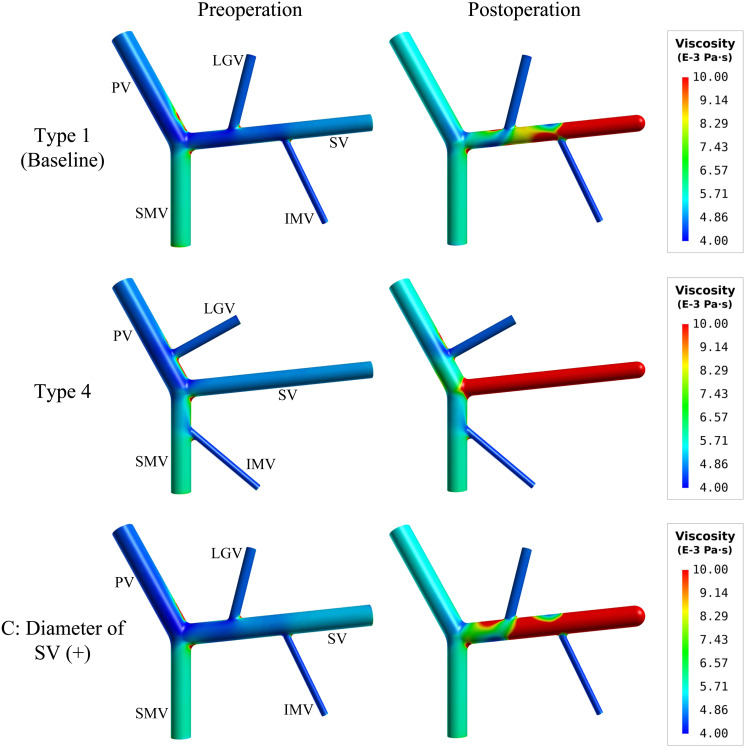
Spatial distributions of simulated blood viscosity in the near-wall regions of the baseline (type 1) model, type 4 model, and the type 1 model with the increase of the SV diameter [i.e., ‘C’(+) variation].

Figure 7 further shows the spatial distributions of simulated blood viscosity in the near-wall regions of three representative models [i.e., type 1 model (baseline), type 4 model, and type 1 model with ‘C’ (+) variation (diameter of SV (+))]. The simulated blood viscosity was overall low in all preoperative models, but increased significantly after splenectomy, especially in the SV where the maximal value of viscosity was increased by over 100% compared to the preoperative value. In the meantime, inter-model differences in blood viscosity were remarkably enlarged after splenectomy. Recalling the distributions of WSS shown in Figures 3, 4, one can easily find that the distributions of low WSS correspond well with the distributions of high viscosity. This can be explained by the viscosity model described by Eq. 1 in which blood viscosity is inversely related to flow shear rate, which makes blood viscosities in regions with low WSS and shear rate higher than those in regions with high/normal WSS and shear rate.

## Discussion

While postoperative thrombosis is a frequent surgical sequela in patients treated with splenectomy (Ikeda et al., 2005; Ushitora et al., 2011) and numerous studies have been devoted to exploring its etiology and associated factors (Sobhonslidsuk and Reddy, 2002; Winslow et al., 2002; Ruiz-Tovar and Priego, 2016; Tsamalaidze et al., 2018), well-established schemes for patient-specific risk assessment remain absent in current clinical practice. In the context, we devised a computational model-based study to investigate in detail the influences of anatomical and morphogeometrical variations of the PVS, which are common in the population, on hemodynamic conditions in the PVS before and after splenectomy. In particular, we focused on identifying anatomical and morphogeometrical features associated with the wall area ratio of low WSS (ALS) in light of the fact that low WSS has been well demonstrated to be an important driving factor for thrombosis (Malek et al., 1999; Poredos and Jezovnik, 2018). Major findings from our study include: (1) anatomical or morphogeometrical variations of the PVS induced considerable changes in the characteristics of WSS distribution and flow patterns both before and after splenectomy, but had little influence on ALS before splenectomy, (2) after splenectomy, ALS increased significantly and became more sensitive to variations in the anatomical structure and morphogeometrical parameters of the PVS, and (3) blood viscosity increased significantly after splenectomy, especially in regions with low WSS.

The low ALS before splenectomy indicates that hemodynamic conditions in the PVS are basically preventive of the formation of thrombus irrespective of inter-patient differences in anatomical or morphogeometrical features and associated hemodynamic characteristics, which may explain the clinically observed low incidence of thrombosis in portal hypertensive patients not treated with splenectomy (Ikeda et al., 2005; Parikh et al., 2010; Ushitora et al., 2011). Before splenectomy, the abundant blood perfusion from the spleen dominates hemodynamic characteristics in the SV, keeping blood flow velocity high, and the flow field stable and resistant to disturbances caused by mingling flows from the tributaries (see Figures 3, 5). However, the situation was substantially altered after splenectomy. The absence of blood flow from the spleen after splenectomy not only remarkably raised ALS, causing the preoperatively clot-preventive hemodynamic conditions in the PVS to convert into clot-promoting conditions, but also rendered flow field in the SV highly dependent on blood perfusion from the tributaries, and more sensitive to anatomical or morphogeometrical variations. Specifically, the type 4 anatomical structure (with connections of IMV-SMV and LGV-PV) was found to undergo the largest increase in ALS following splenectomy (from preoperative 0.86% to postoperative 35.19%), while the type 1 anatomical structure (with connections of IMV-SV and LGV-SV) had the lowest postoperative ALS (21.28%). The postoperative ALS of the type 1 structure increased to 29.39% when incorporating the ‘C’(+) variation (i.e., increasing the diameter of SV by 2.625 mm), and to 25.12% when incorporating the ‘D’(−) variation (i.e., shortening the distance between the IMV and splenoportal junction by 20 mm). In addition, introducing curvature to the SV in the type 1 structure with ‘D’(+) variation led to an evident increase of ALS (from 15.79% to 23.56%). Relatively, changes in ALS induced by varying the angles between tributary veins and their trunk veins were much smaller and nearly negligible in comparison with the aforementioned ALS changes induced by other anatomorphological variations. These results indicate that the anatomical structure and some morphogeometrical parameters (e.g., the diameter and curvature of the SV, and the distance between the IMV and splenoportal junction) of the PVS can significantly affect postoperative ALS and associated risk of postsplenectomy thrombosis. The marked increase in postoperative ALS associated with ‘C’(+) variation may provide biomechanical evidence for explaining the clinical observation that patients with a larger diameter of the SV were at increased risk of developing thrombosis after splenectomy (Danno et al., 2009; Kinjo et al., 2010; de’Angelis et al., 2017; Huang et al., 2018). However, clinical studies on the correlations between the anatomical structure or other morphogeometrical features of the PVS and the risk of postsplenectomy thrombosis remain absent. At this point, our findings may serve as a theoretical reference for guiding future clinical studies in this direction, or for roughly assessing the risk of postsplenectomy thrombosis based on medical images of the PVS available in general clinical settings.

Moreover, viscosity is one of the blood rheological properties that affect thrombus formation. High blood viscosity has been found to increase the risk of thrombosis (Dormandy and Edelman, 1973). In this study, we represented the non-Newtonian rheology of blood with the Carreau model in all hemodynamic simulations, finding that blood viscosity was overall increased after splenectomy, with high viscosity having similar spatial distributions and showing similar sensitivities to morphogeometrical variations of the PVS as low WSS. In a sense, increased blood viscosity may play an additive role to low WSS in promoting the formation of thrombus.

While the present study provided useful theoretical insights for assessing the risk of postsplenectomy thrombosis based on the anatomorphological features of the PVS, the findings must be considered in the context of several limitations. First, we kept flow velocities assigned to the inlets of the tributary veins unchanged before and after splenectomy. However, flow rates in the tributary veins may increase after splenectomy, probably as a consequence of compensatory responses to the surgery, although relevant clinical data have not been reported in the literature. Based on the measured change ratio (30%) of flow rate in the PV before and after splenectomy (Bai et al., 2020) and the population-averaged ratio (45%) of flow rate in the SV to that in the PV, we estimate that the overall postoperative compensatory increase in flow rates in the tributary veins might be within 30%. To clarify whether such postoperative compensatory increase in flow rate would have great influence on postoperative ALS, we performed an additional numerical simulation by increasing flow rates in all tributary veins in the Type 1 model by 30%. The results showed that a 30% increase in flow rates only had slight influence on postoperative ALS, causing ALS to decrease from 21.28 to 19.10%. In this sense, a moderate compensatory increase in flow rates in the tributaries of the PVS may have secondary influence on postoperative ALS compared to the anatomorphological features of the PVS. Second, for purpose of simplicity, we only incorporated morphogeometrical variations into the baseline anatomical structure (i.e., type 1). It remains unclear whether the findings would be applicable to other types of anatomical structure. Our additional numerical simulations revealed that incorporating the ‘C’(+) variation into the other three anatomical types or incorporating the ‘D’(−) variation into the type 2 structure also led to considerable increases in ALS, although the increases in ALS were quantitatively different from those observed in the case of type 1 structure, and that the negligible effects on ALS of varying the angles between tributary veins and PV, SV or SMV remained (results not shown). For the type 3 and type 4 structures, changing the distance of the IMV relative to the splenoportal junction had little influence on ALS since the IMV is connected to the SMV where the values of WSS are much higher than the threshold value of low WSS. Third, our numerical studies with respect to the hemodynamic effects of anatomical and morphogeometrical variations have been performed in a one-factor-at-a-time way, however, the anatomical and morphogeometrical features of the PVS in a real patient may contain multiple factors addressed in the present study whose combined influences on postoperative hemodynamic conditions and ALS could be highly complex. In this sense, patient-specifically quantifying hemodynamic conditions and ALS through image-based model reconstruction and hemodynamic analysis would be necessary to better predict the risk of postsplenectomy thrombosis.

## Data Availability Statement

The original contributions presented in the study are included in the article/supplementary material, further inquiries can be directed to the corresponding author/s.

## Author Contributions

TW, FL, and ZZ: study concept and design. TW and FL: computational modeling, numerical simulation, and data analysis. TW: drafting of the manuscript. FL and ZZ: critical revision of the manuscript. All authors contributed to the article and approved the submitted version.

## Conflict of Interest

The authors declare that the research was conducted in the absence of any commercial or financial relationships that could be construed as a potential conflict of interest.
